# Microbial plankton configuration in the epipelagic realm from the Beagle Channel to the Burdwood Bank, a Marine Protected Area in Sub-Antarctic waters

**DOI:** 10.1371/journal.pone.0233156

**Published:** 2020-05-27

**Authors:** Valeria A. Guinder, Andrea Malits, Carola Ferronato, Bernd Krock, John Garzón-Cardona, Ana Martínez

**Affiliations:** 1 Instituto Argentino de Oceanografía (IADO), Consejo Nacional de Investigaciones Científicas y Técnicas (CONICET), Bahía Blanca, Argentina; 2 Centro Austral de Investigaciones Científicas (CADIC), Consejo Nacional de Investigaciones Científicas y Técnicas (CONICET), Ushuaia, Argentina; 3 Alfred-Wegener-Institut Helmholtz-Zentrum für Polar- und Meeresforschung, Bremerhaven, Germany; 4 INQUISUR (UNS-CONICET) Departamento de Química, Universidad Nacional del Sur UNS, Bahía Blanca, Argentina; University of Connecticut, UNITED STATES

## Abstract

Marine microbial plankton hold high structural and functional diversity, however, high-resolution data are lacking in a large part of the Global Ocean, such as in subpolar areas of the SW Atlantic. The Burdwood Bank (BB) is a submerged plateau (average depth 100 m) that constitutes the westernmost segment of the North Scotia Ridge (54°–55°S; 56°–62°W). The BB hosts rich benthic biodiversity in low chlorophyll waters of the southern Patagonian Shelf, Argentina, declared Namuncurá Marine Protected Area (NMPA) in 2013. So far, the pelagic microorganisms above the bank have not been described. During austral summer 2016, we assessed the microbial plankton (0.2–200 μm cell size) biomass and their taxonomical and functional diversity along a longitudinal transect (54.2–55.3°S, 58–68°W) from the Beagle Channel (BC) to the BB, characterized by contrasting hydrography. Results displayed a marked zonation in the composition and structure of the microbial communities. The biomass of phytoplankton >5 μm was 28 times higher in the BC, attributed mainly to large diatom blooms, than in oceanic waters above the BB, where the small coccolithophore *Emiliania huxleyi* and flagellates <10 μm dominated. In turn, the biomass of microheterotrophs above the BB doubled the biomass in the BC due to large ciliates. Notably, toxic phytoplankton species and their phycotoxins were detected, in particular high abundance of *Dinophysis acuminata* and pectenotoxins above the bank, highlighting their presence in open subpolar regions. Picophytoplankton (<2 μm), including *Synechococcus* and picoeukaryotes, were remarkably important above the BB, both at surface and deep waters (up to 150 m). Their biomass surpassed by 5 times that of phytoplankton > 5 μm, emphasizing the importance of small-sized phytoplankton in low chlorophyll waters. The homogeneous water column and high retention above the bank seem to favor the development of abundant picophytoplankton and microzooplankton communities. Overall, our findings unfold the plankton configuration in the Southern Patagonian Shelf, ascribed as a sink for anthropogenic CO_2_, and highlight the diverse ecological traits that microorganisms develop to adjust their yield to changing conditions.

## Introduction

Biodiversity is a key ecological trait of terrestrial and marine environments, regarded as an important regulator of ecosystem productivity, functioning and resilience, and further defines the ecosystem sustainability and services [1,2]. Marine microbial plankton hold high diversity of unicellular life forms and play key roles in biogeochemical cycles, food webs and climate regulation [3,4,5]. These microorganisms encompass prokaryotes and eukaryotes, and cover a wide size-spectra (pico-, nano- and microplankton, i.e. 0.2 to 200 μm) [6]. The composition and structure of microbial communities are reliable indicators of hydro-climatological conditions at wide spatio-temporal scales due to fast responses attributed to their small size, short-term life cycles and passive displacement [7,4,8]. Moreover, they exhibit different ecological traits and trade-offs under changing environments, such as resource requirements, feeding modes, size, morphology, mobility, and toxin production [9,10]. This functional diversity is overlooked when bulk chlorophyll is used as a proxy of phytoplankton biomass and productivity [11], and hence signatures of structure and functionality are increasingly considered in global models [12,13,14].

As the foundation of marine food web, planktonic microorganisms modulate the trophic networks and the carbon flows between the pelagic and benthic realms [15,16], which are in turn affected by vertical stratification and mixing, as well as zooplankton grazing [17,5] and viral lysis [18,19]. The Southern Ocean [20] and in particular the extensive Patagonian shelf are sinks of atmospheric CO_2_ [21,22,23]. This has been mostly related to the dominance of diatoms, dinoflagellates and coccolithophores in productive areas such as mid-shelf and shelf-break fronts in spring and summer [24,25,26,27]. Furthermore, several studies highlight the importance of smaller phytoplankton in summer production, i.e. pico- and nanoplankton in the Southern Patagonian Shelf [28], in other areas of the Southern Ocean (e.g. [17]) or in other high latitude marine environments [29,30], moreover as global climate changes [31,32].

Considering the crucial importance of microorganisms in marine ecosystems, surveys of high resolution plankton diversity are essential to assess environmental changes and underlying drivers. This is of particular concern in wilderness and protected areas of the Global Ocean [33], such as the underexplored Pacific-Atlantic connection, the Beagle Channel (BC), and the Namuncurá Marine Protected Area (NMPA) at the Burdwood Bank (BB), a submarine plateau 200 km eastern from the Staten Island (Isla de Los Estados), in Sub-Antarctic waters. The BC and the southern Patagonian Shelf are productive systems [28,34,35] influenced by the Cape Horn Current which transports low-salinity waters from the Southeast Pacific, and by the Antarctic Circumpolar Current (ACC) that transports nutrient-rich waters into the Argentine Shelf [36]. The BC and surrounding areas sustain a considerable portion of marine secondary production, including commercial crustacean species such as the king crabs (*Lithodes santolla* and *Paralomis granulosa*) and fish species [37,38]. Likewise, the oceanic NMPA holds high benthic biodiversity, particularly invertebrates with high endemism for this area [39], including cold water corals [40,41] and Asteroidea [42]. A recent study highlighted the key role of brachiopods in providing benthic refuge, food and substrate for other organisms in the NMPA [43]. Further, the area provides spawning and nursery habitat for important commercial fish species, such as the Patagonian sprat (*Sprattus fuegensis*) and the Patagonian toothfish (*Dissostichus eleginoides*) [44,45,46]. Nevertheless, few studies on phytoplankton [47,34] and microzooplankton [48] exist for the BC and even less offshore towards open oligotrophic waters [49], and none above the BB. Along the transition from the BC to the BB, complex hydrographical processes result from the convergence of different water masses and the irregular bathymetry [50,36], that lead to contrasting sub-regions likely inhabited by different plankton assemblages.

Here we assess the microbial plankton structure in terms of species composition, functional types, size fractions, carbon biomass, feeding modes and phycotoxin profiles across a longitudinal transect from the BC to the BB during summer 2016. We aim to contribute to the baseline knowledge of regional biodiversity, as well as to the global biogeography of key species such as toxic plankton. In addition, we aim to characterize the microbial plankton configuration under contrasting hydrological conditions, and ultimately to bring light to the potential carbon sources above the BB that may fuel the benthic communities. Overall, our high resolution plankton study in an underexplored subpolar area of the SW-Atlantic extends the database for global-scale studies on changing ocean conditions.

### Study area

**Fig 1:**
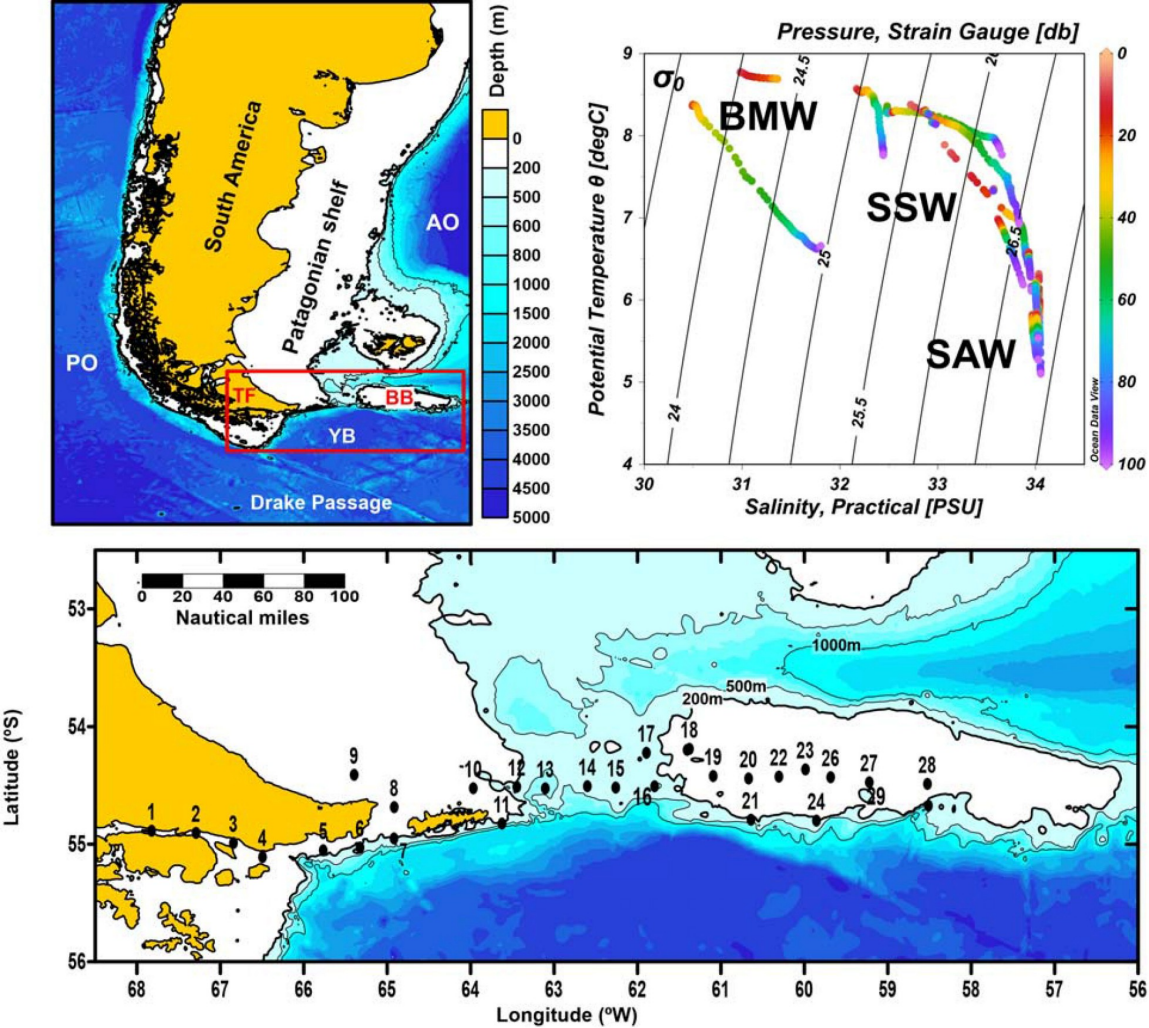
Study area at the south of the Argentine Patagonian shelf, SW Atlantic. In the lower panel, the 29 sampling stations of the BOPD cruise in December 2016 are shown. Bathymetric data: IOC, IHO, and BODC (2003). PO = Pacific Ocean, AO = Atlantic Ocean, TF = Tierra del Fuego, YB = Yaghan Basin, BB = Burdwood Bank. In the temperature-salinity diagram, the acronyms indicate the water masses: Beagle Magellan Water (BMW), Subantarctic Shelf Water (SSW) and Subantarctic Water (SAW).

At the southern extreme of South America (≈55°S), the Beagle Channel (BC) connects the Pacific and the Atlantic Oceans through Tierra del Fuego Archipelago (**Fig 1**). The convergence of Antartic and Sub-Antartic waters confers high hydrological complexity to this area, interconnected with the Cape Horn Current (CHC) that enters the continental shelf through the Le Maire Strait in the eastern part of Tierra del Fuego, and the Antarctic Circumpolar Current (ACC) [50,36]. The high nutrient load entering the Argentine shelf from the south through the ACC is partly diluted by the freshwater input from glacier melting through the BC. In addition, the shelf area receives the influence of continental runoff along the west coast of southern Patagonia, and discharges of urban and industrial effluents in the channel [51,52,35]. The Burdwood Bank (BB) is a shallow but wide (~34,000 km^2^) seamount located around 200 km eastern from the Staten Island (**Fig 1**), a segment of the North Scotia Ridge in the SW Atlantic Ocean. The BB is topped by a plateau with an average depth of 100 m and hills of only 50 m depth at some locations. Below the 200 m isobath, the declivity of the seafloor increases sharply and in fact the bank is bordered at its southern side by depths of more than 4000 m in the Yaghan Basin (Drake Passage) (**Fig 1**) [40]. Two branches of the ACC contour the bank along its eastern and western sides and merge north of the bank to flow equatorward along the Argentine shelf break as the Malvinas/Falkland Current [53,36,54]. A part of the BB (~28000 km^2^) has been declared a Marine Protected Area (MPA) since 2013 and named Namuncurá (Law 26875, Argentina), based on its species richness and strategic location for climatological research, following international criteria for vulnerability and conservation [55].

## Materials and methods

### Field sampling, CTD

An oceanographic expedition was carried out on board the R/V Puerto Deseado (BOPD, CONICET-Argentina) during the beginning of the austral summer, from 7 to 17 December 2016. This expedition was part of the National Strategic Initiative, Pampa Azul (http://www.pampazul.gob.ar/), that aims to assess the biodiversity and functioning of the NMPA. The field study of microbial plankton did not involve endangered or protected species. Along a longitudinal transect of ~1100 km (54.1–55.2° S, 58–68° W), 29 sampling stations were covered for plankton study (**Fig 1**). The sampling encompassed the BC and the continental shelf (stations 1 to 10), the transition zone (stations 11 to 17), defined as the passage between the Staten Island and the western edge of the BB, and the BB (stations 18 to 29). At each station, vertical profiles of seawater temperature and conductivity, and fluorescence of chl-*a*, were acquired by means of a CTD SBE-25 supplemented with a Seapoint fluorometer. The data were further processed and derived variables (salinity, density, depth) obtained with the SBE data processing software version 7.23.2. Further, the Brunt–Väisäla (N^2^) parameter of water column stability was calculated applying the equation: N^2^ = —(g ∂ρ/ρ_o_ ∂z), where g = gravity of the Earth (9.8 m^2^ s^−1^), ρ = density of water, and z = depth. The variables were plotted using the software Ocean Data View, version ODV 4.7.10.

### Chlorophyll *a* and microbial plankton >5 μm

Surface samples (3–5 m depth) were collected using Niskin bottles for plankton quantification (cell size >5 μm) and chlorophyll *a* (chl-*a*) estimations. Samples were not available at stations 13 and 15. For plankton analysis, 250 mL were fixed with Lugol’s solution (1% final concentration) and kept in dark until the examination under microscopy. For the analysis of the chl-*a* concentration, a volume of 200–250 mL was filtered through a glass fiber filter (Whatman GF/F) and filters stored at -20°C until pigments were extracted with 90% acetone for 24 h in the darkness at 4°C. Chl-*a* was then quantified by fluorimetry after Holm-Hansen et al. [56] with a Shimadzu RF-5301 at Ex/Em: 460/671 nm, using pure chl-*a* from *Anacystis nidulans* as standard according to Arar and Collins [57]. In addition, water samples from the upper 20 m of the water column were collected by vertical net tows (20 μm mesh), and fixed with formaldehyde (2% final concentration) for qualitative analyses of plankton.

For plankton quantification, subsamples of 50–100 mL were settled in Utermöhl composite sedimentation chambers during 24 h, and cells >5 μm were enumerated using a Wild M20 inverted microscope according to Hasle [58]. The entire chamber was analyzed under magnification of 400 x. Plankton abundance was then expressed in cells L^-1^. For biomass estimation (in μC L^-1^), cell dimensions were measured throughout the counting procedure using an ocular micrometer. Thereafter, plankton cell volumes (in μm^3^) were calculated assigning simple geometric shapes to species according to Hillebrand et al. [59] and transformed into carbon content (pg C cell^-1^) using two different carbon-to-volume ratios, one for diatoms and one for all the other planktonic groups [60]. Species identification was performed on net haul samples under a Zeiss Standard R microscope and a Nikon Eclipse microscope, using phase contrast, differential interference contrast (DIC) and magnification of 1000 x. Local and regional references (*e*.*g*. [47,48,25]) as well as general literature of plankton taxonomy (*e*.*g*. [61,62]) were consulted.

### Picoplankton

Water samples for picoplankton (0.2–2 μm) abundance analysis were collected using Niskin bottles mounted on a rosette from 3 to 5 depths per station, down to 200 m depth. Subsamples for heterotrophic bacteria (1 mL) and picophytoplankton (5 mL) were fixed with 0.2 μm-pre-filtered glutaraldehyde (0.5% and 0.1% final concentration for bacteria and picophytoplankton, respectively), incubated for 15–30 min at 4°C, subsequently flash-frozen in liquid nitrogen and stored at -80°C until analysis [63]. Afterwards, the samples were thawed, bacteria were stained with SYBRGreen I (Molecular Probes Inc.) for 10 min in the dark and quantified in a Becton Dickinson FACSCalibur flow cytometer after dilution with TE buffer (10 mM Tris, 1 mM EDTA, ph = 8) as described previously [64]. For picophytoplankton enumeration, unstained samples were analyzed in a Becton Dickinson FACSCalibur flow cytometer at high speed (*ca*. 100 μL min^-1^) following Marie et al. [63]. Phototrophic populations (*Synechococcus* and picoeukaryotes) were discriminated according to their light scatter and specific autofluorescence properties. The sample flow rate was accurately calibrated following the protocol by Marie et al. [63] and used to calculate *in situ* abundances for bacteria and picophytoplankton. Cell abundances for picoplankton were converted to carbon biomass assuming a carbon content of 20 fg C cell^-1^, 255 fg C cell^-1^ and 2590 fg C cell^-1^ for heterotrophic bacteria, *Synechococcus* and picoeukaryotes, respectively [65,66].

### Phycotoxins

Plankton samples were collected by three vertical net tow hauls from 30 m depth to surface with a 20 μm-mesh of 40 cm diameter (7–9 Dec.; stations 4–7, 11–14, 16 and 17) and 23 μm mesh size (10–15 Dec.; stations 1–3, 8–10, 19–24, 26–29). Net haul concentrates were pooled and adjusted to a defined volume of 0.4–1.8 L (depending on the net tow volume) with 0.2 μm-filtered seawater. An aliquot of 100 mL was fixed with acidic Lugol’s iodine solution (1% final concentration) for species identification. The rest was collected on a 20 μm mesh and rinsed with little filtered seawater into a 50 mL centrifugation tube and then adjusted to a final volume of 50 mL. A10 mL aliquot was fixed with Lugol’s solution. The remaining 40 mL were split into four aliquots, and each aliquot was centrifuged at 3,500 x g in a 15 mL centrifugation tube (model 2036, Rolco, Buenos Aires, Argentina). The supernatants were discharged, pellets resuspended in approximately 1 mL filtered sea water and transferred to a 2 mL cryovial and centrifuged again (10 min, 3220 x g, model 5415 D, Eppendorf, Hamburg, Germany). Finally, supernatants were removed and cell pellets frozen at -20°C.

Cell pellets for the analysis of paralytic shellfish poisoning (PSP) toxins were resuspended in 500 μL 0.03 M acetic and for lipophilic toxins in 500 μL methanol and subsequently homogenized with 0.9 g of lysing matrix D by reciprocal shaking at maximum speed (6.5 m s^-1^) for 45 s in a Bio101 FastPrep instrument (Thermo Savant, Illkirch, France). After homogenization, samples were centrifuged at 16,100 x g at 4°C for 15 min. The supernatants were transferred to spin-filters (0.45 μm pore-size, Millipore Ultrafree, Eschborn, Germany) and centrifuged for 30 s at 800 x g, followed by transfer to autosampler vials.

Analysis of multiple lipophilic toxins was performed by liquid chromatography coupled to tandem mass spectrometry (LC-MS/MS), as described in Krock et al. [67]. Toxin contents are expressed as nanograms per net tow (ng NT^-1^). Paralytic shellfish poisoning toxins were analyzed by ion-pair chromatography coupled to post-column derivatization and fluorescence detection (PCOX method) as described in detail in Van de Waal et al. [68].

### Data analysis of microbial plankton >5 μm

Cluster analysis was applied to the species’ abundances in the sampling stations to assess the spatial structure of the plankton community. The abundance (in cells L^-1^) was log-transformed to normalize data. A matrix of similarities between each pair of sampling station was calculated using the Bray-Curtis similarity index. Afterwards, the species that were revealed by SIMPROF test in the cluster analysis, were pointed out in the map. The organisms with higher contribution to differences among groups were identified by means of SIMPER (Similarity Percentages) test. Statistical analysis was performed using the software PRIMER [69].

The surface water temperature, salinity, chl-*a*, and water column stability N^2^ assessed by means for the upper 100 m, were compared in the three zones: the BC and the continental shelf, the transition zone, and the BB, by means of T-tests. The means for each zone were estimated using the individual stations as replicates.

## Results

### Water column structure

**Fig 2:**
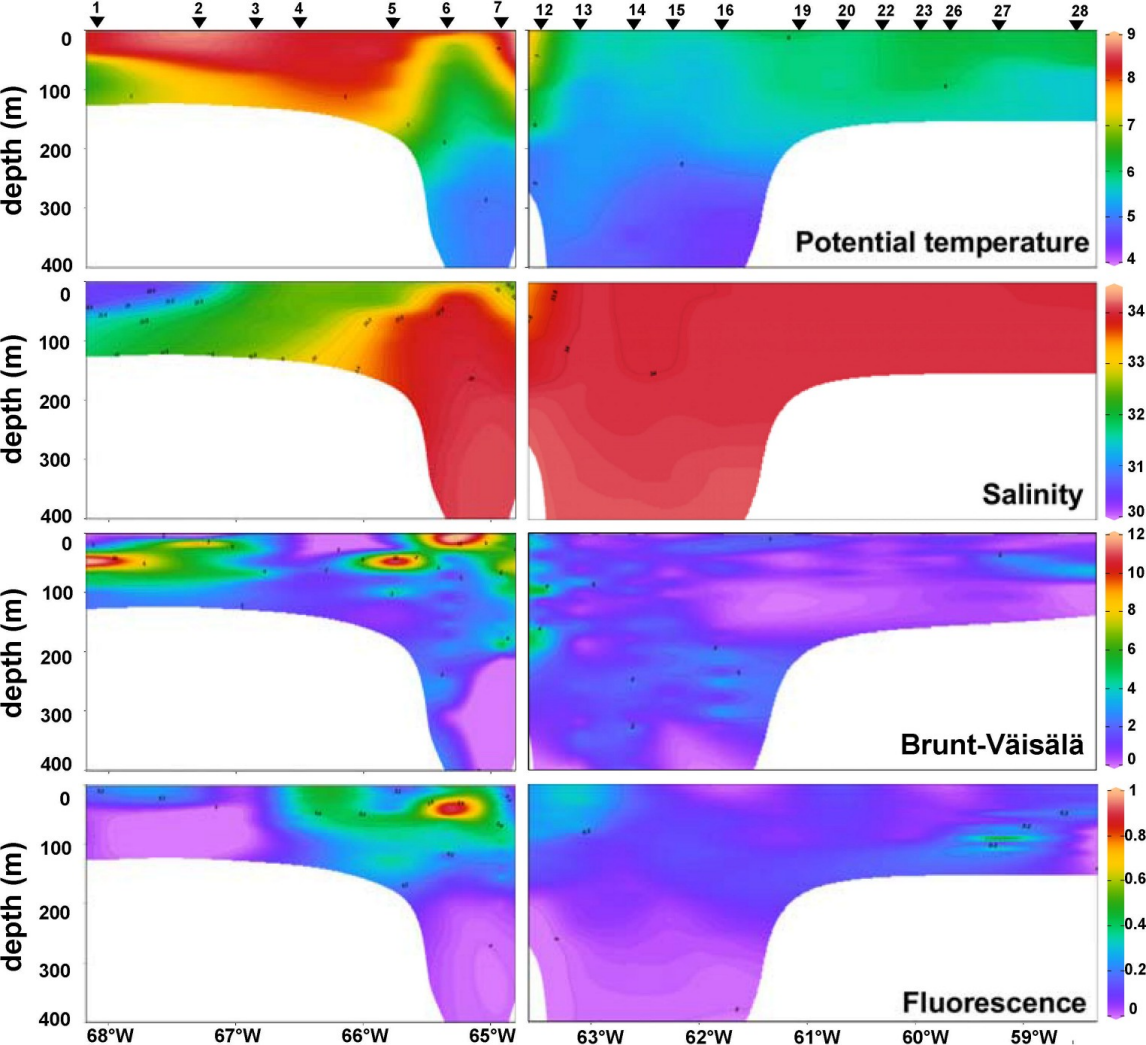
Vertical profiles of physical parameters. Potential temperature (°C), salinity, Brunt Wäisälä parameter of water column stability (cyc h^-1^) and fluorescence (RFU, relative fluorescence unit). The parameters are shown in two transects: from the Beagle Channel to the eastern Staten Island (left panels, stations 1 to 7) and from the Western Staten Island to the Burdwood Bank (right panels, stations 10 to 28). Stations 8, 9, 17, 18 and 29 are not included in these transects.

**Fig 3:**
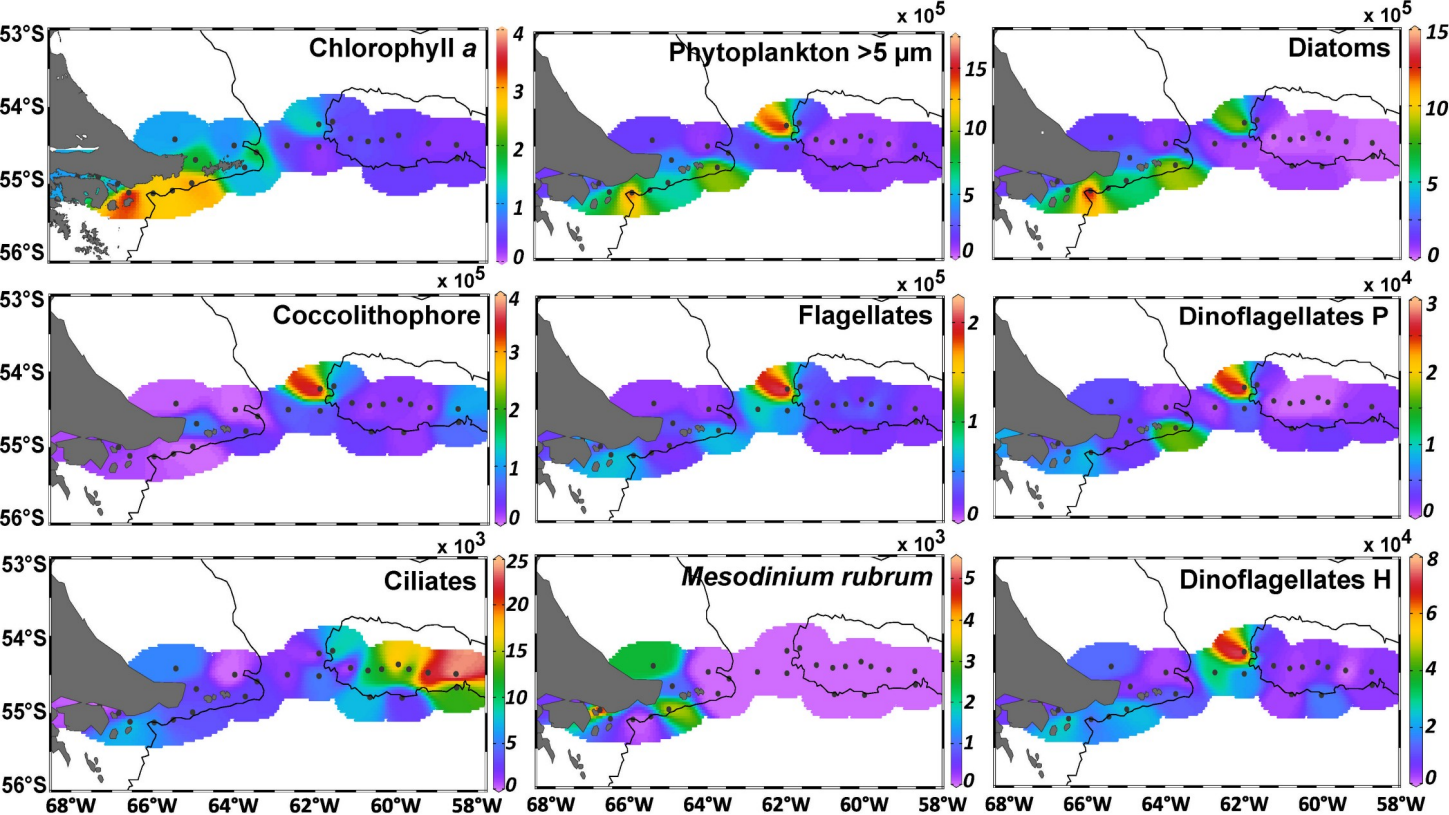
Surface *in situ* chlorophyll *a* concentration (μg L^-1^) and abundance (cells L^-1^) of microbial plankton > 5μm. Phytoplankton includes diatoms + flagellates + coccolithophore *(Emiliania huxleyi*) + phototrophic dinoflagellates (dinoflagellates P). The three bottom panels are heterotrophic protists: ciliates, *Mesodinium rubrum* (a mixotrophic ciliate which is an obligate prey of the toxic *Dinophysis acuminata*) and dinoflagellates H.

Different water masses were depicted in the T-S diagram (**Fig 1**) following an increase in salinity and decrease in temperature from West to East: Beagle Magellan waters (BMW), Subantarctic Shelf Waters (SSW) and Subantarctic Waters (SAW). The vertical profiles (**Fig 2)** showed that the water column gradually shifted from stratified in the BC and the shelf area east of Staten Island, towards homogeneous waters across the transition zone and above the BB. Further, fluorescence was maxima on the shelf, especially at stations 4 to 7 above ~40 m depth (**Fig 2**), where a large diatom bloom was detected (**Fig 3**), and then decreased towards the bank. In particular, station 17 did not match the described trend, because of rather high fluorescence and relative high surface salinity, displaying a slightly stratified profile (see Discussion and **Fig 11**). Likewise, stations 27 and 28 above the bank showed an increase of fluorescence from surface to 100 m depths.

Regarding surface water conditions (< 5 m depth) in the three zones across the longitudinal transect, SST and salinity were (mean ± standard deviation) 8.71 ± 0.43°C and 32.33 ± 0.99 in the BC and shelf area (stations 1 to 10), 6.85 ± 0.95°C and 33.54 ± 0.35 in the transition zone (stations 11 to 17), and 6.58 ± 0.17°C and 33.88 ± 0.02 in the BB (stations 18 to 29). Statistical comparisons (T-Test) showed that SST and salinity in the BC were significantly different to those in the BB (p<0.001, n = 22, and p<0.0001, n = 22, respectively). In turn, the transition zone was different from the BC in terms of SST (p<0.001, n = 17) and salinity (p<0.005, n = 17, respectively), but not significantly different from the BB (p = 0.31, n = 19 and p = 0.26, n = 19, respectively). The water column stability assessed by means of the Brunt-Väisälä parameter (N^2^) for the upper 100 m was higher (p<0.001, n = 17) in the BC-shelf area (5.16 ± 0.38 cyc h^-1^) than in the transition (1.63 ± 0.45 cyc h^-1^) and the BB (1.07 ± 0.36 cyc h^-1^, p<0.001, n = 22), but it was not different between the transition and the BB (p = 0.34, n = 19).

### Distribution of chlorophyll and microbial plankton >5 μm

**Fig 4:**
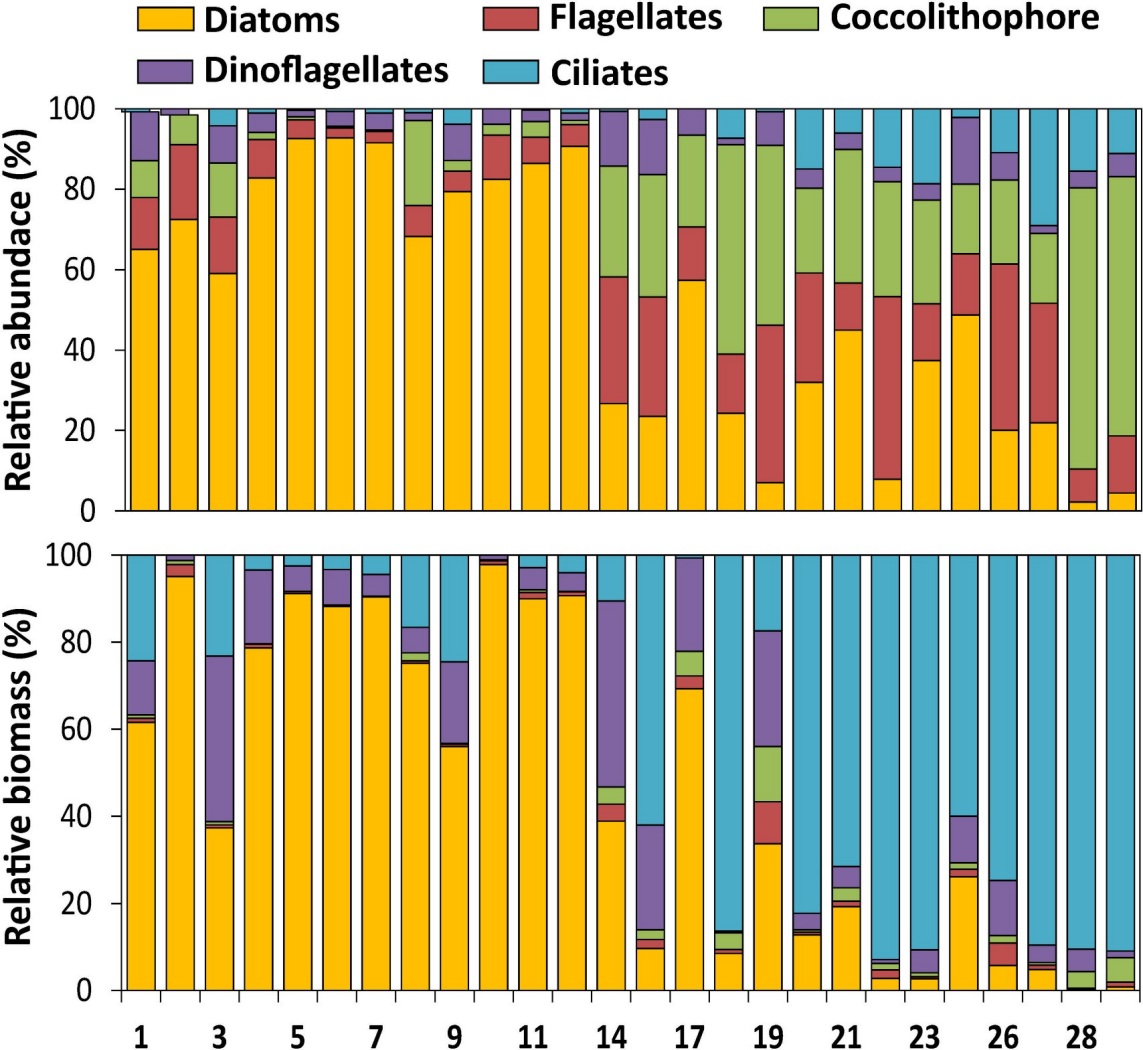
Relative abundance (cells L^-1^, upper panel) and biomass (μg C L^-1^, lower panel) of planktonic groups (> 5 μm) expressed in % at the sampling stations.

The surface *in situ* chl-*a* concentration and the total phytoplankton abundance (cell size >5μm) showed decreasing trends from the shelf to the BB (**Fig 3**), with the exception of station 17 that displayed a remarkably different plankton assemblage. T-Test showed that chlorophyll was higher in the BC (1.9 ± 1.03 μg L^-1^) with respect to the BB (0.41 ± 0.09 μg L^-1^, p<0.0005, n = 21) and the transition zone (1.02 ± 0.64 μg L^-1^, p = 0.04, n = 15), but not different between the BB and the transition zone (p = 0.15, n = 16). Maximal values of surface chlorophyll (> 2 μg L^-1^) were detected at stations 4 to 7 due to a phytoplankton bloom of up to 14.7 x 10^5^ cells L^-1^ at station 5. This bloom was dominated by large diatoms (>85% of total phytoplankton assemblage, **Figs 3 and 4**), and in general, diatoms were the most frequent group in the BC-shelf area (stations 1 to 12), representing more than 50% of the total abundance and carbon biomass (**Fig 4**). Likewise, at station 17, the phytoplankton reached the maximal abundance of 15.6 x 10^5^ cells L^-1^ (**Figs 3 and 4**), but the biomass was relatively low (**Fig 4**), indicating the dominance of small species (see the following section). Other phytoplankton groups were coccolithophores, represented by a single species: *Emiliania huxleyi*, flagellates and dinoflagellates, the three of them with the maximal abundances at station 17 (**Fig 3**). In terms of relative abundance, the coccolithophore and the flagellates were more frequent in the transition area and the BB (from station 14 to 29, **Fig 4**), but their contribution to carbon biomass was rather negligible (**Fig 4**) due to their small cell sizes (*E*. *huxleyi* 3–8 μm and flagellates 5–15 μm).

Regarding the microheterotrophs, ciliates were more abundant above the BB (**Figs 3**and **4**), where large species dominated and their contribution to carbon biomass was significant (**Fig 4**). Abundance of heterotrophic dinoflagellates was rather conservative along the transect, with maximum at station 17 (**Fig 3**), followed by relatively high biomass in the shelf at stations 4 to 7 (**Fig 4**) together with the diatom bloom.

### Spatial configuration of microbial plankton >5 μm

**Fig 5:**
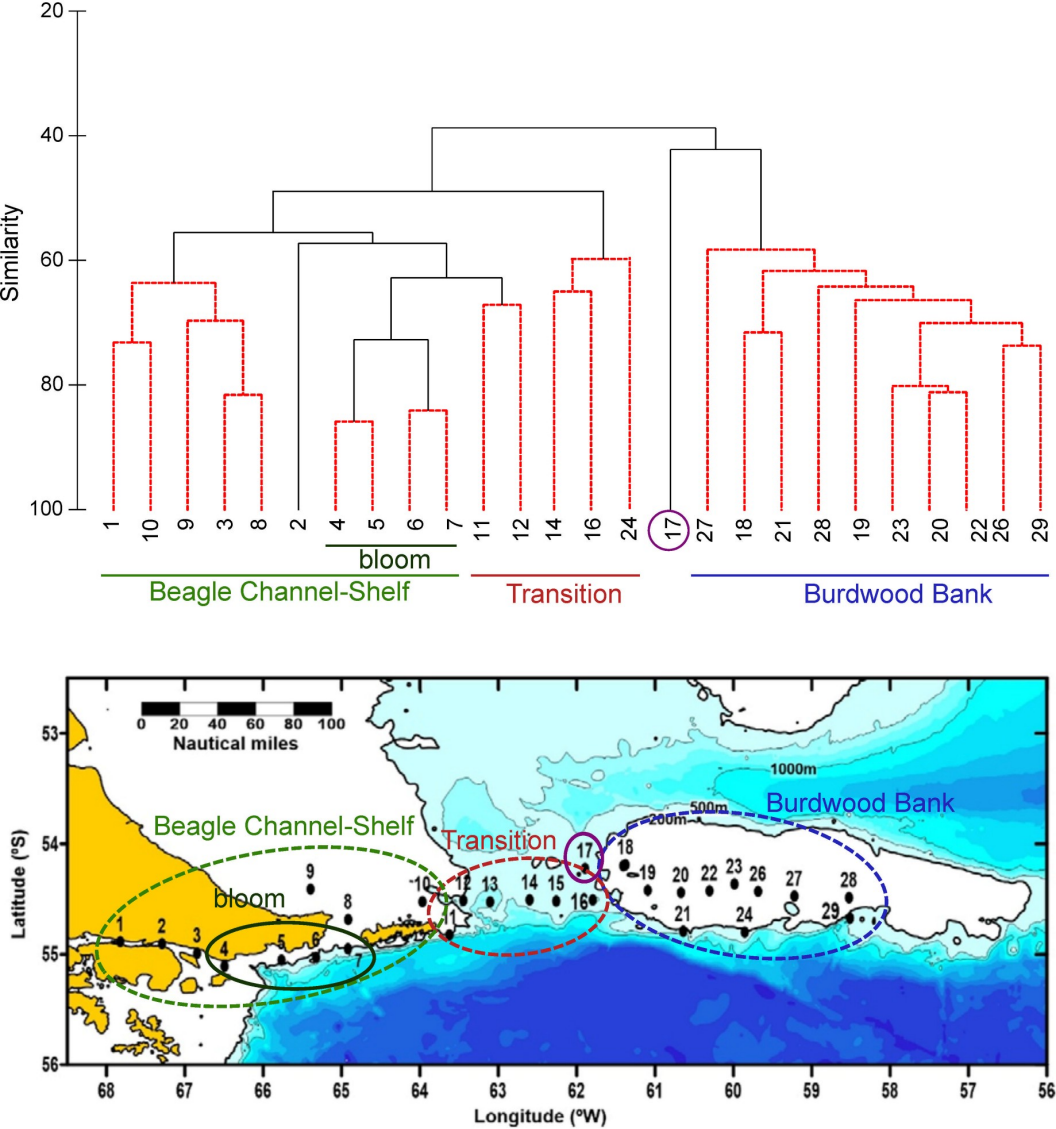
Plankton assemblages. **(A)** Cluster analysis based on Bray Curtis similarity index of the microbial plankton > 5 μm, at the study stations during the expedition in the austral summer. The abundance of the species (cells L^-1^) were transformed using log (x+1). **(B)** The results of the cluster analysis are shown in the map of the study area.

**Fig 6:**
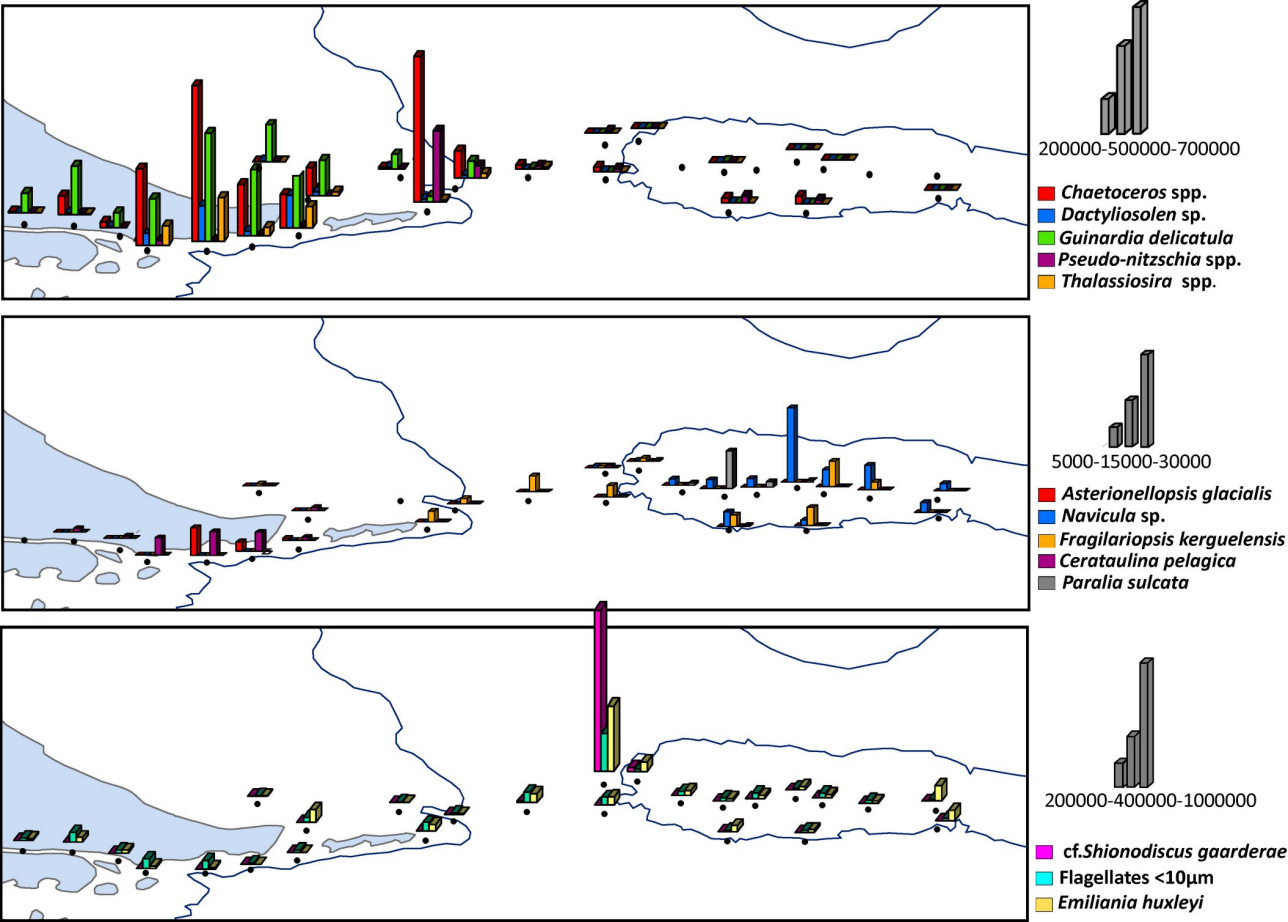
Most representative species of phytoplankton > 5 μm responsible of the spatial zonation resulted from SIMPER analysis. Panels **(A)** and **(B)** are diatoms, **(C)** shows the diatom cf. *Shionodiscus gaarderae*, the coccolithophore *Emiliania huxleyi* and nanoflagellates.

The cluster (**Fig 5**) and SIMPER analyses (**Fig 6**) depicted a clear zonation based on different plankton assemblages along the study transect. Three main areas emerged: (1) the BC-shelf area, from stations 1 to 10, (2) the transition area, from station 11 to 16, and (3) the BB area, from station 18 to 29. In particular, station 17 represented a separated group due to its notably different plankton community (**Figs 5 and 6**), and a subgroup arouse inside the BC-shelf area embracing stations 4 to 7, here called bloom area (**Fig 5**). All the identified species in the cruise are detailed in S1 Table.

**(1) BC-shelf area:** large diatoms were the dominant phytoplankton group in terms of diversity and abundance, especially in the blooming area (**Fig 6**). Phototrophic dinoflagellates that characterized this area belong to the armored genera cf. *Azadinium*, *Prorocentrum*, *Alexandrium*, *Ceratium* and *Oxytoxum*. Among the heterotrophic dinoflagellates, *Gymnodinium* spp. were the most frequent, followed by *Protoperidinium* spp., *Gyrodinium* spp, *Amphidinium* and *Torodinium robustum*. The mixotrophic ciliate *Mesodinium rubrum* (obligate prey of the potentially toxic dinoflagellate *Dinophysis*) was relatively frequent and only present in this area (**Fig 3**). Small aloricate ciliates (< 25 μm) were dominated by the genera *Strombidium*, *Strombidinopsis* and *Strobilidium*, as well as species of loricate ciliates from the genus *Tintinnopsis*.

**(2) Transition area:**Phytoplankton was mainly represented by small flagellates and the coccolithophore *Emiliania huxleyi* (**Fig 6**). Station 17, in particular, was dominated by a bloom of a small (9–15 μm) diatom species: *cf*. *Shionodiscus gaarderae* (up to 9.2 x 10^5^ cells L^-1^, equivalent to 98% of total diatoms at the station) (**Fig 6**). No particular dinoflagellate or ciliate species arose in this area, except for heterotrophic unarmored dinoflagellates, *Gymnodinium* spp. and *Gyrodinium* spp.

**(3) BB area:** The phytoplankton of this area was mainly represented by small flagellates -the silicoflagellate *Dictyocha speculum* was conspicuous- and the coccolithophore *E*. *huxleyi*. In a lesser extent, a few diatoms were present (**Fig 6**). Dinoflagellates were dominated by large heterotrophic species of *Gymnodinium* and *Gyrodinium*, where *Gyrodinium spiralis* was significantly important. Ciliates were highly diverse and dominated by large species (>30 μm) of the genera *Strombidium*, *Strombidinopsis*, *Strobilidium* and *Cyrtostrombidium*, and the large species *Laboea strobila* and *Strombidium conicum* were remarkably important.

### Picoplankton distribution

**Fig 7:**
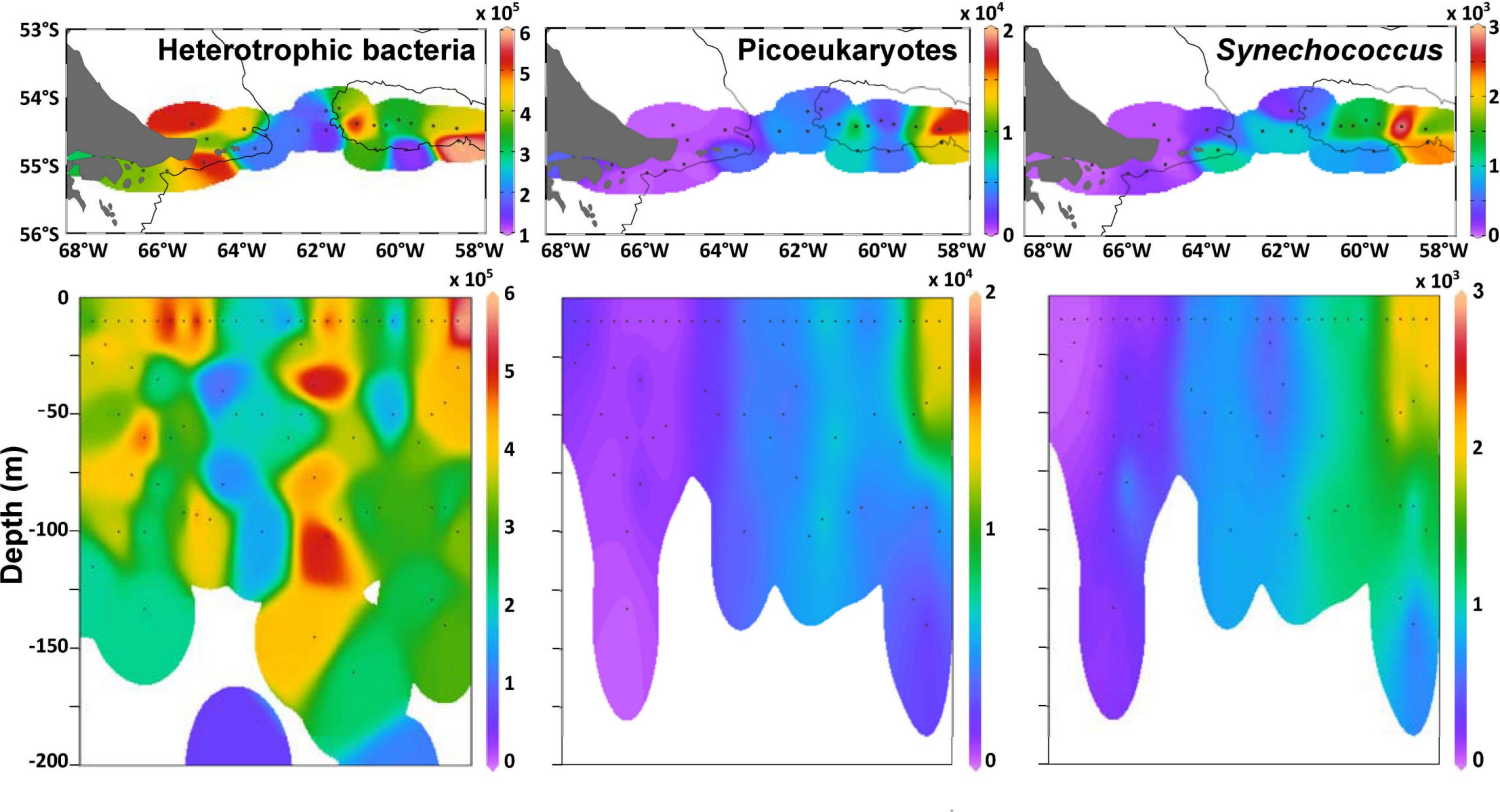
Distribution of picoplankton (in cells L^-1^) in the surface **(A)** and in the water column **(B)**.

**Fig 8:**
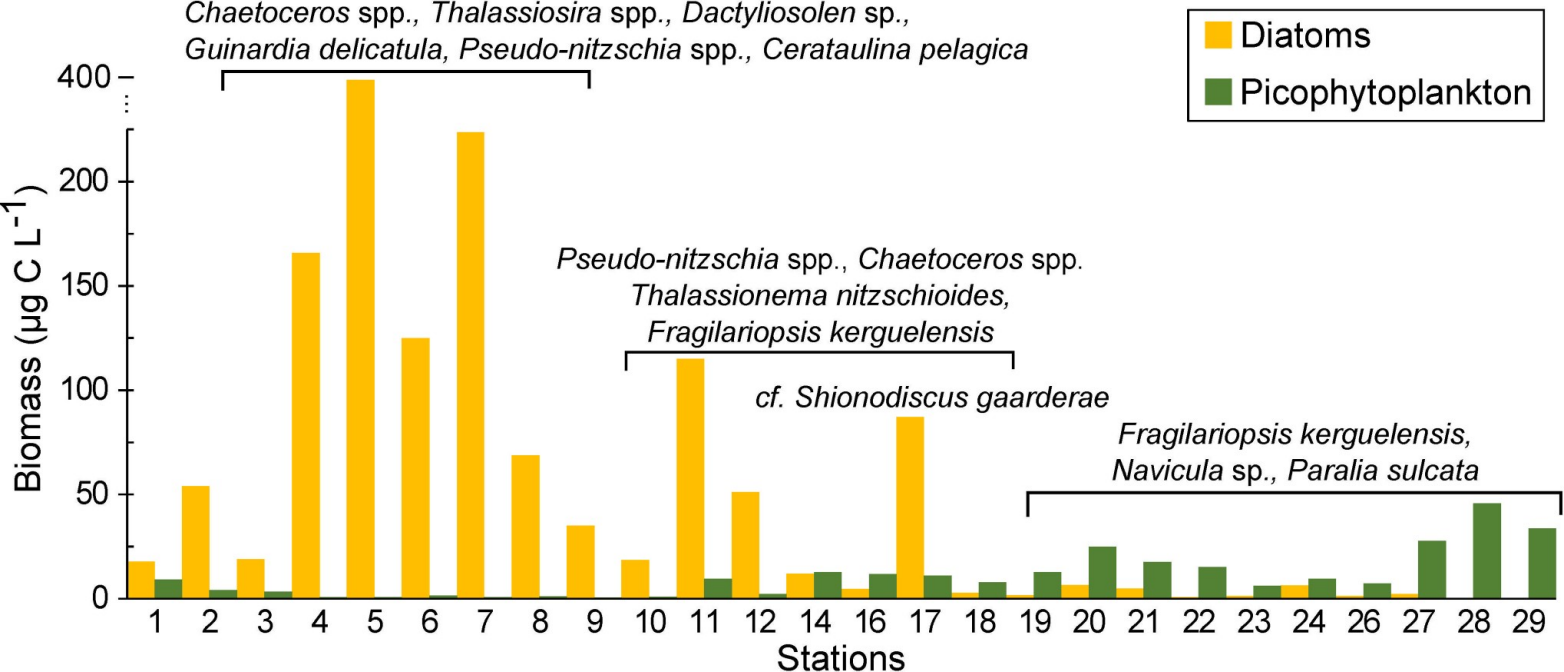
Surface biomass (carbon content) of diatoms and picophytoplankon (picoeukaryotes + *Synechococcus*) along the sampling stations. The diatom species that contributed most to the biomass in each area are indicated.

The abundance of heterotrophic bacteria ranged from 1–6 x 10^5^ cells L^-1^ along the transect, being highest (> 3 x 10^5^ cells L^-1^) in the BC-shelf and in the BB areas, while in the transition zone it was consistently lower (< 2 10^5^ cells L^-1^) (**Fig 7**). The biomass of heterotrophic bacteria (7.2 ± 2.5 μg C L^-1^) was rather low compared with the biomass of the picophytoplankton, with a conservative behavior along the transect. Regarding the phototrophic picoeukaryotes and the cyanobacteria *Synechococcus*, both displayed noticeable maxima above the BB, from surface waters to ~75 and 150 m depth, respectively (**Fig 7**). In terms of carbon content, the biomass of picophyoplankton (picoeukaryotes+ *Synechococcus*) was 5 times the biomass of phytoplankton >5 μm above the BB (station 18 to 29), and around 7 times the biomass of diatoms, as shown in **Fig 8**.

### Phycotoxins and associated potential producers

**Fig 9:**
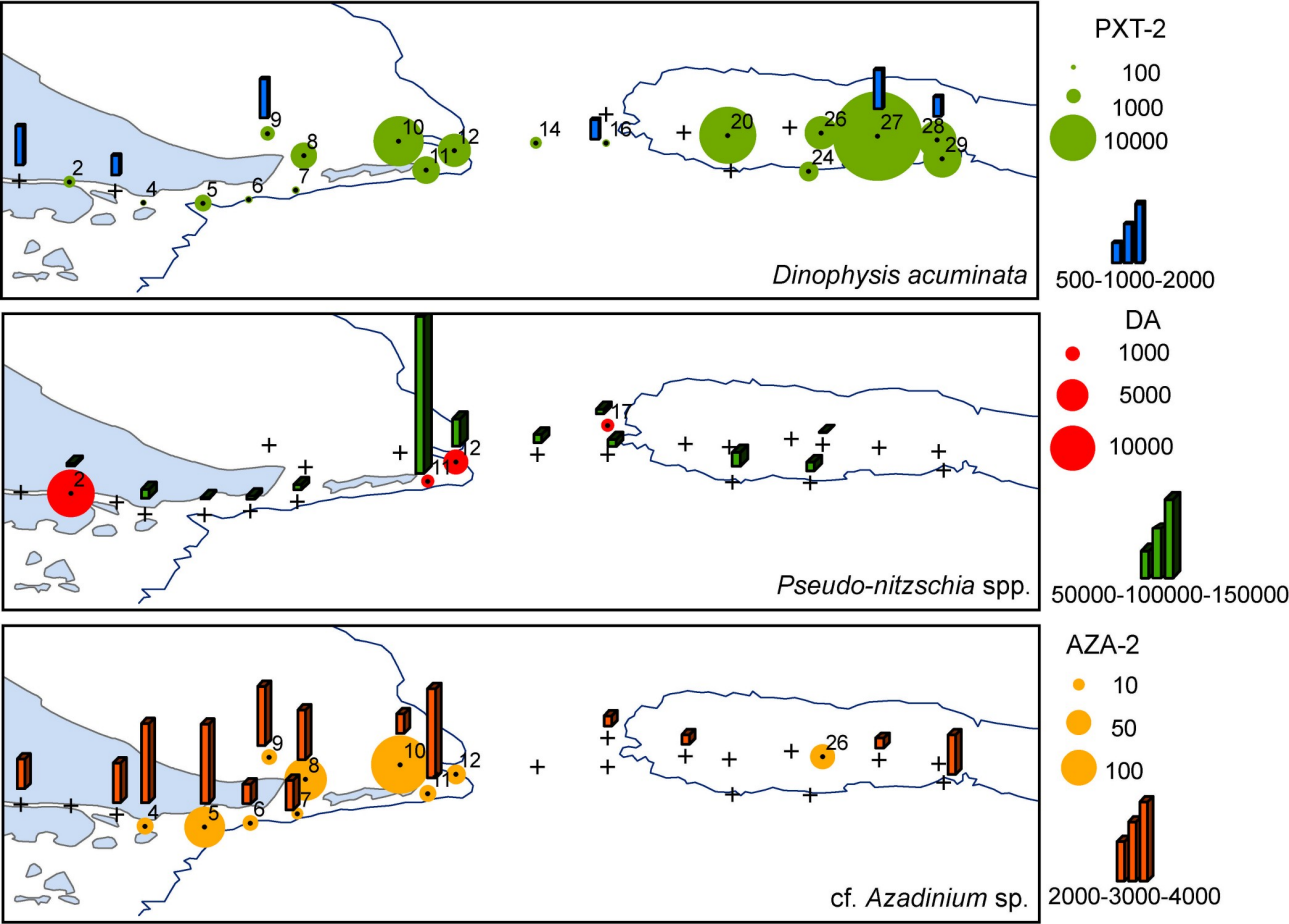
Surface distribution of phycotoxins (circles, in ng NT^-1^) and the potential planktonic producers (bars, in cells L^-1^). AZA-2: azaspiracids, DA: domoic acid and PTX-2: pectenotoxins. Only the stations where toxins were detected are indicated with numbers.

Among the toxins detected during the survey were azaspiracid-2 (AZA-2) produced by small dinoflagellates of the genus *Azadinium*, pectenotoxin-2 (PTX-2) produced by several species of the genus *Dinophysis*, and domoic acid (DA) produced by species of the diatom genera *Nitzschia* and *Pseudo-nitzschia* (**Fig 9**). The least abundant toxin was AZA-2, with a maximum value of 130 picograms per net tow (pg NT^-1^) at station 5, whereas all other values ranged between 5 and 65 pg NT^-1^. AZA-2 was almost exclusively found in the area between the outer BC and Staten Island with the exception of trace levels of 12 pg NT^-1^ detected at station 26 above the BB. Generally, *Azadinium* spp. and AZA-2 co-occurred at most stations (**Fig 9**). The least widely distributed toxin was DA, which was found at abundances between 805 pg NT^-1^ at station 11 and 2,970 pg NT^-1^ at station 12 and at stations 2 and 17 at intermediate levels (**Fig 9**). The geographic range of DA was similar as that of AZA-2 ranging from BC to Staten Island. Larger numbers of *Pseudo-nitzschia* cells were only detected at neighboring stations 11 and 12 co-occurring with DA. In contrast, *Pseudo-nitzschia* cells were detected at some stations without DA and vice versa (**Fig 9**). The most abundant toxin found during the survey was PTX-2 at levels between 280 and 9,100 pg NT^-1^ with a maximum level of 13,625 pg NT^-1^ at station 20. In contrast to AZA-2 and DA, PTX-2 was hardly detected in BC, but appeared at higher amounts around the eastern tip of Staten Island and above the BB (**Fig 9**). Interestingly, *Dinophysis* and PTX-2 only co-occurred at stations 27 and 28. The only species of this dinoflagellate genus detected during the survey was *Dinophysis acuminata*.

## Discussion

### Microbial plankton configuration

**Fig 10:**
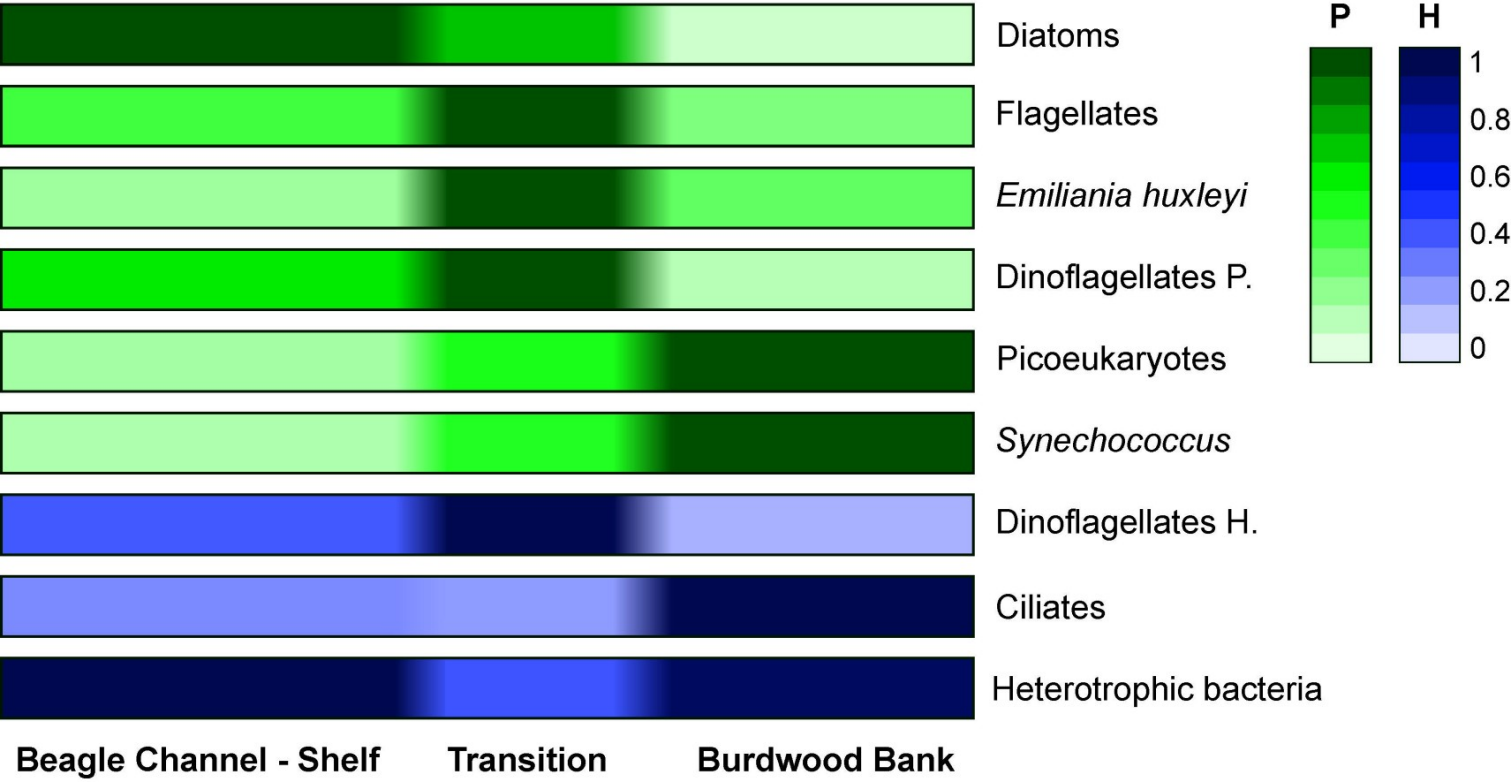
Normalized averaged densities (cells L^-1^) of the microbial plankton groups in the three areas depicted with the cluster analysis. The BC-shelf area includes stations 1 to 10, the transition zone includes stations 11 to 16, and the BB encompasses stations 18 to 29. Green and blue colors correspond to phototrophic and heterotrophic modes, respectively.

The Sub-Antarctic region of the Southern Patagonian Shelf plays a key role in the sequestration of atmospheric carbon through microbial activity, and this study contributes to the scarce data of the microbial plankton in the area to assess the ecosystem functioning. The biodiversity characterization also enriches the global datasets of species distribution and their ecological repertoire under different hydrological settings. In this sense, distinctive zonation was disclosed in the structure and composition of the plankton community from the Beagle Channel (BC) to the Burdwood Bank (BB) during the sampling period in early summer (**Fig 10 and S1 Table**). The water column gradually shifts from nutrient-rich, more turbid (e.g. [35]) and stratified in the channel and shelf, towards vertically homogeneous above the bank. The Chl-*a* in surface layers, a proxy of phytoplankton biomass, decreased along this gradient, with maximal values in coastal and shelf waters (up to 3.6 μg L^-1^) and minimal values above the bank (<0.5 μg L^-1^). On a monthly basis, Almandoz et al. [19] found marked seasonality in phytoplankton biomass in coastal areas in the BC during the period 2015–2016, with the lowest values in autumn-winter (average 0.92 μg L^−1^) and the highest in spring-summer (average 2.36 μg L^−1^). As in our study, their highest chl-*a* peaks (5.0 to 5.5 μg L^−1^) were associated with diatom blooms, mainly represented by *Chaetoceros* and *Thalassiosira* species. Likewise, Garzón Cardona et al. [35] also described a decreasing trend in phytoplankton biomass (from 2.9 to 0.2 μg L^−1^) in summer 2012 from the BC towards oceanic waters, following the increasing salinity and decreasing trends in particulate organic carbon (POC) and nitrogen (PON), and dissolved organic matter (DOM) [35,70]. Despite there are no references on the nutrient levels above the BB, a recent high-resolution circulation model [54] pointed out that mixing and upwelling driven by tides and the ACC, are important processes above the bank, and they may supply micronutrients from deep waters to fertilize primary productivity beyond the surface layers.

In our study, the environmental gradients also delineated the phytoplankton niches and trophic regimes. Therefore, a mix of large, chain-forming and fast growing diatom species was blooming on the shelf (e.g., *Chaetoceros*, *Thalassiosira*, *Pseudo-nitzschia*, *Dactyliosolen*, *Guinardia*), as they are superior competitors under high nutrients and relatively low underwater light conditions. This is supported by previous observations in this Patagonian cold estuarine zone [71,70,47], and overall in estuarine areas (e.g. [72]) and other high latitude coastal systems (e.g. [73,74]). Conversely, in open waters above the bank, few diatom species were present, e.g. *Fraguilariopsis kerguelensis*, *Navicula* sp. and *Paralia sulcata*, characterized by slow-growth and heavily silicified walls. Large parts of the Southern Ocean are high-nutrient low chlorophyll (HNLC) areas, mainly due to the co-limitation of light and micronutrients such as iron [75]. Hence, the diatom species distribution is in agreement with the hypothesis of iron availability on the control of carbon export to the deep ocean, that poses that in iron-enriched areas, lightly silicified diatoms with rapid population growth and uptake of nutrients dominate, ascribed as carbon-sinkers. In contrast, in iron-depleted zones, diatoms with opposite skills are common, regarded as siliceous-sinkers [4]. Moreover, in surface layers above the bank, the small haptophyte *Emiliania huxleyi* and phytoflagellates (<10 μm) were growing successfully. This may be related to the large surface-to-volume ratio of small phytoplankton cells that confers them superior competitive abilities for nutrient uptake in open sea waters that are commonly nutrient-depleted areas [76]. In light of climate-driven ocean warming and strengthen vertical stratification, this may have important implications for the microbial plankton size configuration [77,36]. It is worth noting that cells of the haptophyte *Phaeocystis* (< 5 μm) were observed in this expedition, especially in the BC, but no quantification was performed under microscopy. Almandoz et al. [19] documented exceptional blooms (up to 15–17 x 10^6^ cells L^-1^) of *Phaeocystis* cf. *antactica* in spring 2015 in the channel. Regarding the key role that haptophytes play in the regulation of the climate system [78] and their particularly high abundance in Sub-Antarctic [24] and Antarctic waters (e.g. [74,79]), monitoring their distribution may provide insights of climate driven ecosystem changes.

Phototrophic picoeukaryotes and the cyanobacteria *Synechoccocus* were the largest contributors to microbial carbon biomass above the BB. Furthermore, at some stations, picophytoplankton was abundant up to 150 m deep, suggesting a trophic connection with the shallow benthic communities on the BB. Again, this high carbon biomass in deeper waters may be due to mixing and energetic and persistent uplifting of nutrient-rich deep ocean waters [54], which may enhance the phytoplankton production beyond the photic layers. In turn, microheterotrophs are the key link between the smallest primary producers and the mesozooplankton, and in fact, large ciliates contributed with high abundance and carbon biomass above the bank. Growing evidence supports that the smaller phytoplankton fractions play key roles in trophic networks and carbon export in oceanic waters, like the pivotal importance of the picoplankton taxon *Synechococcus* (e.g. [80,15]). The exportation of small cells to the sea floor may occur by aggregation or by zooplankton grazing and sinking of their pellets [80,5], enhanced by the homogeneous water column and high water mass retention above the bank. In agreement with other studies in the Southern Ocean [81,17], our findings seem to contrast the axiom that large phytoplankton mediates the carbon transfer to the sea floor, and highlight the importance of small cells-based plankton food web in low chlorophyll waters of the subpolar Atlantic Ocean.

**Fig 11:**
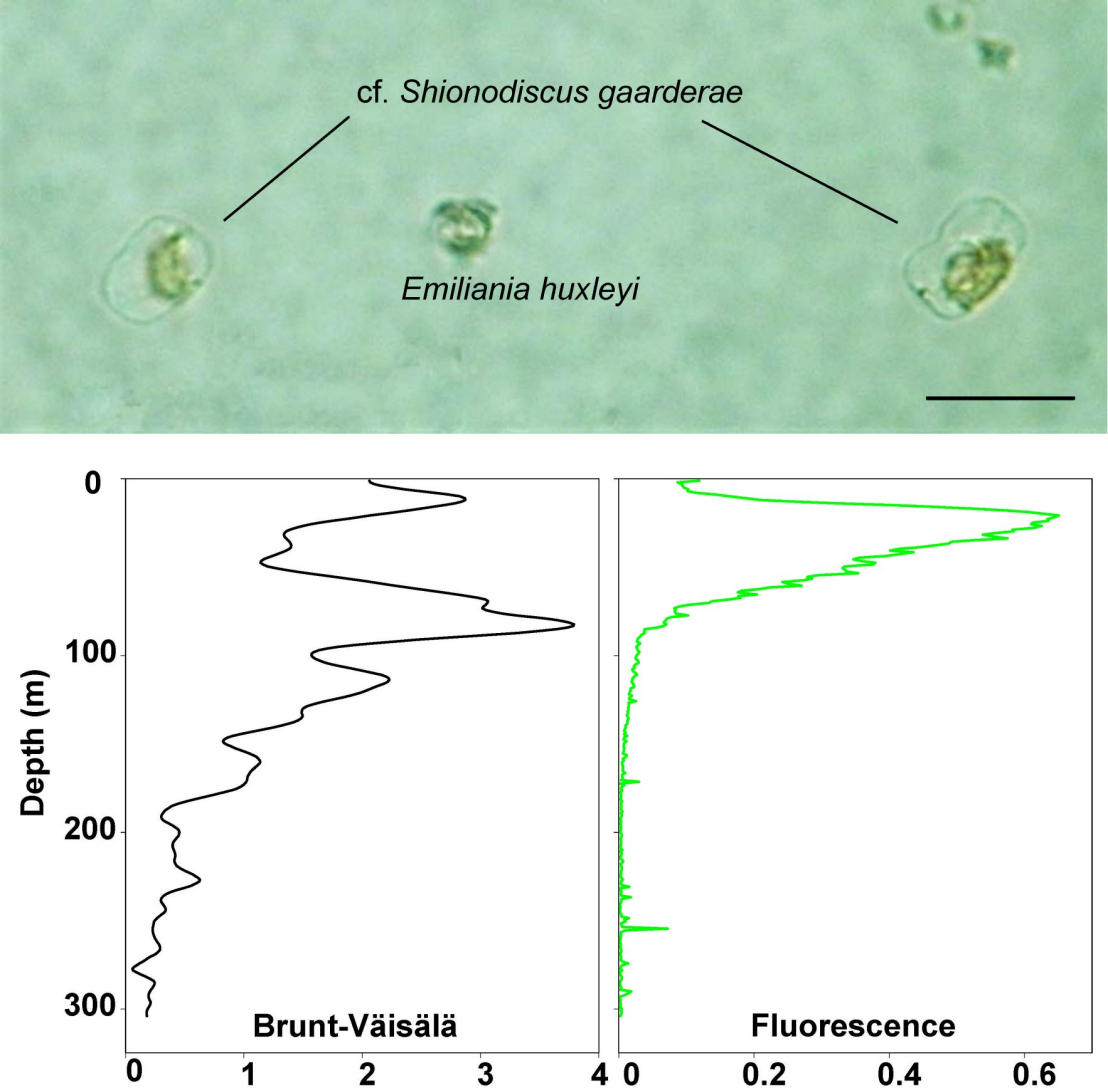
Dominant blooming species and water column structure at station 17. The photography shows two weakly silicified cells of the small diatom cf. *Shionodiscus gaarderae* (biovolume: 2828 μm^3^, carbon content: 181 pg C cell^-1^), and a cell of the coccolithophore *Emiliania huxleyi*. Scale bar: 15 μm. In the lower panels, the vertical profiles of water column stability (Brunt-Väisälä parameter, in cyc h^-1^) and the fluorescence at station 17 are shown.

Interestingly, in the transition zone (stations 11 to 16) between the continental shelf and the BB, the microbial plankton abundance was relatively lower, likely related to the intense circulation of Sub-Antarctic waters northward trough this deeper area. However, station 17, located at the Northwestern edge of BB, showed the highest phytoplankton abundance with a completely different species assemblage. The dominance of the lightly silicified cells of the diatom *cf*. *Shionodiscus gaarderae* (**Fig 11**) suggests a fast population response to triggering factors such as nutrient pulses and stratified conditions. This species has been recently identified and described by Ferrario et al. [82] from specimens collected along the Argentine Sea, where its population was responsible of large blooms in slope waters during springs [82]. In fact, at the particular station 17, the branch of the ACC that surrounds the bank clockwise, creates a small eddy (see Figs 5D and 6B in [54]) before ongoing northwards along the Argentinean shelf break as the Malvinas/Falkland Current. This eddy may cause retention of nutrient-rich waters and vertical stratification (**Fig 11**), and seems to favor the occurrence of localized phytoplankton blooms.

### Toxic plankton and toxins

The number of described toxigenic phytoplankton species is increasing in continental shelfs worldwide [83], but in general, there are still remote areas of the Global Ocean that remain poorly explored. For instance, a few studies have lately documented the prevalence of toxic species in the BC [19,47], after more than two decades of an exceptional harmful algal bloom (HAB) in the BC in summer 1992 caused by the dinoflagellate *Alexandrium catenella*, with severe detrimental impacts on the ecosystem services [84].

However, virtually nothing is known about the occurrence of HAB species and their toxins beyond the shelf and above the BB, what makes this study particularly valuable on the current situation of this ecosystem. The occurrence and abundance of toxigenic phytoplankton is indicative of the risk of HABs formation under future global warming scenarios. In addition, phycotoxins can be used as specific taxonomic markers, because the expression of phycotoxins is very genus- and sometimes even species-specific. The tracking of toxigenic species and their toxins is interesting because toxins can move along the pelagic food chain by protistan predation and do not necessarily co-occur with the producing species, if these have been fed on. Therefore, the presence of toxins in a plankton community indicates a previous presence of the producing species, which broadens the temporal frame in comparison to presence of certain plankton species alone. Moreover, toxigenic species, even though mostly belonging to the group of phototrophic dinoflagellates, often do not co-occur, because they have different environmental requirements. The occurrence of single toxigenic species allows for the determination of particular oceanographic features, which would not become obvious by analysis of plankton groups and thus can be complementary.

In this study, the analysis of phycotoxins and their potential producers showed a spatial zonation in their occurrence. Whereas the toxins azaspiracid-2 (AZA-2) and domoic acid (DA) followed often-described trends and were most abundant in the coastal and shelf region, in contrast, pectenotoxin-2 (PTX-2) was found at highest abundances above the BB. An interesting aspect of the high PTX-2 abundances (as an indicator of *Dinophysis* spp.) above the bank is that HAB species are regarded as mostly coastal phenomena [85]. However, the high levels of PTX-2 in the open ocean waters support the idea that this might be an anthropocentrically biased perception, because most human activities occur in coastal areas (*e*.*g*., mussel aquaculture of *Mytilus chilensis* in the BC) and open ocean waters are rarely screened for phycotoxins. The particular circulation in the BB [54], seems to favor the development of dinoflagellates which are slow-growing and usually outcompeted by diatoms under mixing conditions. Another open question is the low abundance of the mixotrophic ciliate *Mesodinium rubrum*, which is the obligate prey of *Dinophysis* and was found only in the BC and shelf waters but not above the BB. This is an unexpected finding as a co-occurrence of predator and prey could be assumed. However, time series surveys are necessary to corroborate the potential depletion of *Mesodinium* due to grazing by *Dinophysis* and assess other microbial trophic interconnections.

The second most abundant toxin was domoic acid DA, and was found in high abundance at the offshore stations of the continental shelf, together with its producer *Pseudo-nitzschia* spp. Lastly, AZA-2, was detected mainly in the shelf area but in low abundance, in co-occurrence with its potential producer, the small dinoflagellate cf. *Azadinium* sp. The occurrence of AZA-2 in Argentinean waters was not unexpected as AZA-2 producing *Azadinium poporum* recently has been described in El Rincón area, central Argentine shelf [86,87], but the finding of AZA-2 in net tow samples was unexpected, because the producing organisms are much smaller (~10 μm length) [86] than the mesh of the used plankton net (20 μm). The occurrence of AZA-2 in net samples may be due to the transfer of toxins from the producing organisms to higher trophic levels through predation, and in the special case of this study, to the clogging of the net by high abundance of large mucilaginous diatoms. These factors certainly would not lead to a complete transfer of AZA-2 from smaller to >20 μm size fractions, which in turn indicates an underestimation of total AZA-2 present in the study area. These first and preliminary toxin data from a very narrow time window of 10 days in summer highlight the presence of some toxigenic species in the southern Patagonian Shelf and the NMPA, and indicate a potential risk for marine biota and fisheries through the accumulation of these toxins in the food web. Marine Protected Areas provide multiple ecological benefits for the environment and human wellbeing, including resistance and adaptation to climate change [88,33], and therefore the success in their conservation and management relies on our understanding of their biodiversity and functioning.

## Conclusions

Overall this study unveils insights of (1) the microbial plankton configuration in relation to hydrographical settings, particularly for the first time above the BB, (2) the occurence of toxic phytoplankton species in oceanic waters of subpolar regions, and (3) the current state of plankton diversity in a pristine area to track possible alterations driven by climate trends. Our findings open ecological question that are pursued in ongoing and future surveys. This first characterization of the microbial plankton community above the BB highlights that despite low surface chlorophyll waters, the benthic communities may be largely fueled by an abundant small-cells plankton food web. In light of recent circulation simulations, the high carbon biomass of picophytoplankton accumulated beyond the surface layers appears to be enhanced by the homogeneous vertical structure of the water column and the high water mass retention above the bank. Further multidisciplinary research is needed to unfold the biogeochemical and hydrological processes modulating the microbial plankton structure and the potential benthic-pelagic interactions.

## Supporting information

S1 Table List of taxa identified in the cruise onboard BOPD during austral summer (December 2016). BC-S: Beagle Channel and Shelf area corresponds to stations 1 to 10, T: Transition zone embraces stations 11 to 17 and BB: Burdwood Bank includes stations 18 to 29.
(DOCX)
