# Supplementary material for: Microbial plankton configuration in the epipelagic realm from the Beagle Channel to the Burdwood Bank, a Marine Protected Area in Sub-Antarctic waters
Source: PLoS One. 2020 May 27;15(5):e0233156. doi: 10.1371/journal.pone.0233156 (PMC7252610; doi:10.1371/journal.pone.0233156)
Supplement: S1 Table — BC-S: Beagle Channel and Shelf area corresponds to stations 1 to 10, T: Transition zone embraces stations 11 to 17 and BB: Burdwood Bank includes stations 18 to 29. (DOCX) [file pone.0233156.s001.docx]

**Table S1.** List of taxa identified in the cruise onboard BOPD during austral summer (December 2016). BC-S: Beagle Channel and Shelf area corresponds to stations 1 to 10, T: Transition zone embraces stations 11 to 17 and BB: Burdwood Bank includes stations 18 to 29.

| **Diatoms** | **BC-S** | **T** | **BB** |
| --- | --- | --- | --- |
| *Actinocyclus curvatulus* Janisch | x | x |  |
| *Asterionellopsis glacialis* (Castracane) Round | x |  |  |
| *Cerataulina pelagica* (Cleve) Hendey | x |  |  |
| *Chaetoceros affinis* Lauder | x |  |  |
| *Chaetoceros concavicornis* Mangin | x |  |  |
| *Chaetoceros debilis* Cleve | x |  |  |
| *Chaetoceros decipiens* Cleve | x |  |  |
| *Chaetoceros didymus* Ehrenberg | x | x |  |
| *Chaetoceros diadema* (Ehrenmberg) Gran | x |  |  |
| *Chaetoceros lorenzianus* Grunow | x |  |  |
| *Chaetoceros similis* Cleve | x |  |  |
| *Chaetoceros tortissimus* Gran | x |  |  |
| *Chaetoceros* spp. | x |  |  |
| *Coscinodiscus* spp. | x |  | x |
| *Dactyliosolen* sp. | x |  |  |
| *Ditylum brightwellii* (West) Grunow in Van Herurck | x |  |  |
| *Eucampia antactica* (Ehrenberg) A.Mann | x |  |  |
| *Fragilariopsis kerguelensis*(O'Meara) Hustedt |  | x | x |
| *Guinardia delicatula* (Cleve) Hasle | x |  |  |
| *Leptocylindrus minimus* Gran | x |  |  |
| *Navicula* spp. | x |  | x |
| *Nitzschia* spp. | x |  |  |
| *Paralia sulcata* (Ehrenberg) Cleve | x |  | x |
| *Podosira stelliger* (Bailey) Mann |  |  | x |
| *Pseudo-nitschia* spp. | x | x | x |
| *Rhizosolenia setigera* Brightwell | x |  |  |
| *Rhizosolenia* sp. | x | x |  |
| cf. *Shionodiscus gaarderae* Ferrario, Almandoz & Cefarelli |  | x |  |
| *Skeletonema* cf. *costatum* (Greville) Cleve emend. Zingone et Sarno | x |  |  |
| *Tabularia fasciculata* (C.Agardh) D.M.Williams & Round |  | x | x |
| *Thalassionema nitszchioides*(Grunow) Mereschkowsky | x |  | x |
| *Thalassiosira anguste-lineata* (Schmidt) Fryxell & Hasle | x |  |  |
| *Thalassiosira eccentrica* (Ehrenberg) Clave | x |  |  |
| *Thalassiosira pacifica* Gran & Angst | x |  |  |
| *Thalassiosira rotula* Meunier | x | x |  |
| *Thalassiosira hendeyii* Hasle & Fryxell | x |  |  |
| *Thalassiosira* spp. | x |  |  |
| **Prymnesiophytes (Coccolithophorids)** |  |  |  |
| *Emiliania huxleyi* (Lohmann) Hay and Mohler | x | x | x |
| *Phaeocystis* cf. *antarctica* Karsten | x | x | x |
| **Silicoflagellates** |  |  |  |
| *Dictyocha speculum* Ehrenberg | x |  | x |
| **Euglenophytes** |  |  |  |
| *Eutreptia sp.* | x |  |  |
| **Other flagellates** |  |  |  |
| flagellates (5-10 µm) | x | x | x |
| flagellates (10-15 µm) | x | x | x |
| flagellates >15 µm | x | x | x |
| *Leucocryptos* sp. | x | x |  |
| **Dinoflagellates** |  |  |  |
| *Akashiwo sanguinea* (Hirasaka) Hansen & Moestrup | x |  |  |
| *Alexandrium sp.* | x | x |  |
| *Amphidinium crassum* Lohmann | x | x |  |
| *Amphidinium sphenoides* Wülff | x | x |  |
| cf. *Amphidoma* sp. | x | x |  |
| cf. *Azadinium* sp. | x | x | x |
| *Ceratium tripos* (Müller) Nitzsch | x |  |  |
| *Dinophysis acuminata* Claparède & Lachmann | x | x | x |
| *Gymnodinium* spp. | x | x | x |
| *Gyrodinium spirale* (Bergh) Kofoid & Swezy | x | x | x |
| *Gyrodinium* spp. | x | x | x |
| *Katodinium* sp. | x | x |  |
| *Oblea baculifera* Balech ex Loeblich Jr. & Loeblich III | x |  |  |
| *Oxytoxum* sp. | x | x |  |
| *Prorocentrum compressum* (Bailey) Abé ex Dodge | x |  |  |
| *Prorocentrum cordatum* (Ostenfeld) Dodge | x | x |  |
| *Prorocentrum* sp. | x |  |  |
| *Protoperidinium bipes* (Paulsen) Balech | x |  |  |
| *Protoperidinium cepa* (Balech) Balech | x |  |  |
| *Protoperidinium pellucidum* Bergh | x |  |  |
| *Protoperidinium* spp*.* | x | x | x |
| *Scrippsiella* sp. | x |  |  |
| *Torodinium robustum* Kofoid & Swezy | x |  |  |
| *Tripos fusus* (Ehrenberg) Gómez | x | x |  |
| Unidentified naked dinoflagellates | x | x | x |
| Unidentified thecate dinoflagellates | x | x | x |
| **Ciliates** |  |  |  |
| *Cyrtostrombidium* sp. |  |  | x |
| *Laboea strobila* Lohmann |  |  | x |
| *Lohmaniella* sp. | x |  | x |
| *Mesodinium rubrum* Leegaard | x |  |  |
| *Strombidium* spp. | x |  | x |
| *Strombidium conicum* (Lohmann, 1908) Wulff |  |  | x |
| *Strombidinopsis* spp. | x |  | x |
| *Strobilidium* spp. | x |  | x |
| *Tintinnopsis* spp. | x |  |  |
| Ciliates < 20 µm | x |  | x |
| Ciliates 20-30 µm | x | x | x |
| Ciliates > 30 µm | x | x | x |
| **Foraminifera** |  |  | x |
| TOTAL | 76 | 32 | 34 |
